# Chemical stability of artemisinin derivatives

**DOI:** 10.1186/1475-2875-11-S1-P99

**Published:** 2012-10-15

**Authors:** Kristel Van Acker, Marc Mommaerts, Stijn Vanermen, Jolien Meskens, Yvan Vander Heyden, Jacqueline Plaizier-Vercammen

**Affiliations:** 1 Arenco Pharmaceutica NV, Aarschot, B-3200, Belgium; 2 Department of Analytical Chemistry and Pharmaceutical Technology Vrije Universiteit Brussel, Brussels, B-1090, Belgium

## Background

Artemisinin derivatives are the most effective and fast acting antimalarials available. Several studies have demonstrated the presence of fake and bad quality antimalarials on the Asian and African market. Stability of artemisinin derivatives has so far only been partially investigated and it is unclear how much this contributes to the reports of bad quality or substandard antimalarials. We analyzed the stability of the three major artemisinin derivatives (artemether, artesunate and dihydroartemisinin) at three stages: the drug substance itself, the mixture of drug substances and different pharmaceutical formulations in different packaging materials, at different climatic conditions.

## Materials and methods

Drug substance powders and pharmaceutical formulations were stored in separate desiccators with different saturated salt solutions representing different climatic conditions according to the ICH guidelines. The content of the drug substance was determined quantitatively using HPLC - UV methods. Ph. Int. specify and require an assay range between 90 and 110% for the finished product.

## Results

In a first step different active substance powders were compared. Artemether at 40°C (with or without exposure to high humidity) is less stable than artesunate at 40°C without exposure to high humidity, but more stable than artesunate exposed to high humidity. Dihydroartemisinin is clearly the least stable and was therefore not used further in this study.

In a second step mixtures of artemether or artesunate with amodiaquine were compared. Artemether with amodiaquine is as stable as artemether alone while no effect from high humidity is seen. Artesunate with amodiaquine is significantly less stable than artesunate alone and is also very sensitive to high humidity.

Finally monolayer combination tablets showed that stability of artemether and even more for artesunate in these tablets decreases further, probably due to acceleration of degradation by the presence of excipients. The results of the artesunate-amodiaquine monolayer combination were very striking since all artesunate had been degraded after only 6 months. Monolayer artemether-amodiaquine combination tablets (Artenam^®^-Plus) are clearly more stable than the monolayer artesunate-amodiaquine tablets.

Comparison of the artesunate-amodiaquine monolayer tablet with a trilayer artesunate-amodiaquine tablet (Arenax^®^) showed that artesunate in the trilayer tablet is more stable than artesunate in the monolayer tablet.

The shelf-life of the monolayer artemether-amodiaquine tablets and trilayer artesunate-amodiaquine tablets need to be confirmed by real-time studies. Stability of artemether in monolayer artemether-amodiaquine combination tablets is still acceptable after 3 years in two real-time conditions (25°C/60%RH and 30°C/65%RH) in PVC blister.

## Conclusions

A significant difference in stability between the three artemisinin derivatives is seen. Dihydroartemisinin is the least stable while artesunate is the most sensitive to humidity and to the combination with amodiaquine.

Active substance powder mixtures are more stable than combination of powders in tablets, probably due to acceleration of degradation by the presence of excipients.

Artesunate and amodiaquine can only be used in a fixed dose combination if they are physically separated.

An artemether-amodiaquine fixed combination tablet does not require this physical separation and is less sensitive to high humidity conditions.

